# Supplementary material for: Magnitude and kinetics of the human immune cell response associated with severe dengue progression by single-cell proteomics
Source: Sci Adv. 2023 Mar 24;9(12):eade7702. doi: 10.1126/sciadv.ade7702 (PMC10038348; doi:10.1126/sciadv.ade7702)
Supplement: Supplementary file 1 — Supplementary Text Figs. S1 to S7 Legends for tables S1 to S6 References [file sciadv.ade7702_sm.pdf]

Science Advances
AAAS

Supplementary Materials for

Magnitude and kinetics of the human immune cell response associated with severe dengue progression by single-cell proteomics

Makeda L. Robinson *et al.*

Corresponding author: Shirit Einav, seinav@stanford.edu

Sci. Adv. **9**, eade7702 (2023)
DOI: 10.1126/sciadv.ade7702

The PDF file includes:

Supplementary Text
Figs. S1 to S7
Legends for tables S1 to S6
References

Other Supplementary Material for this manuscript includes the following:

Tables S1 to S6

Supplementary text:

Challenges in identifying DENV-infected cells in patient-derived PBMCs via mass cytometry

The precise immune cell targets of DENV in the human blood remain incompletely characterized. Myeloid cell types including monocytes and dendritic cells have been shown to be actively infected with DENV (*98, 99*). However a previous study has shown that B cells can maintain DENV infection (*100*) as well, and we have demonstrated that B cell populations, specifically naïve B cells, harbor the large bulk of viral RNA (*10*). Here, we aimed to further define DENV target cells by conjugating three antibodies targeting the DENV envelope (structural), and NS1 and NS3 (nonstructural) proteins (NS1, NS3). Titration of these anti-DENV antibodies in DENV infected Huh7 cells revealed clear bimodal signal and an inoculum dependent increase in DENV protein marker staining at 48 hours postinfection **(Figure S4B).** However, when used to detect DENV in primary patient-derived PBMCs, we detected a greatly increased noise floor, resulting in a small fraction of cells that stained positive for all three DENV proteins **(Figure S4C).** Moreover, there was no correlation between the expression level of the DENV proteins and DENV RNA level measured via RT-qPCR in patient serum **(Figure S4D).** We speculate that the DENV-specific antibodies bound viral protein debris non-specifically associated with immune cell membranes during cell staining, preventing discrimination of infected cells. Further optimization will therefore be required to detect primary DENV-infected cells in PBMCs via CyTOF.

Figure S1

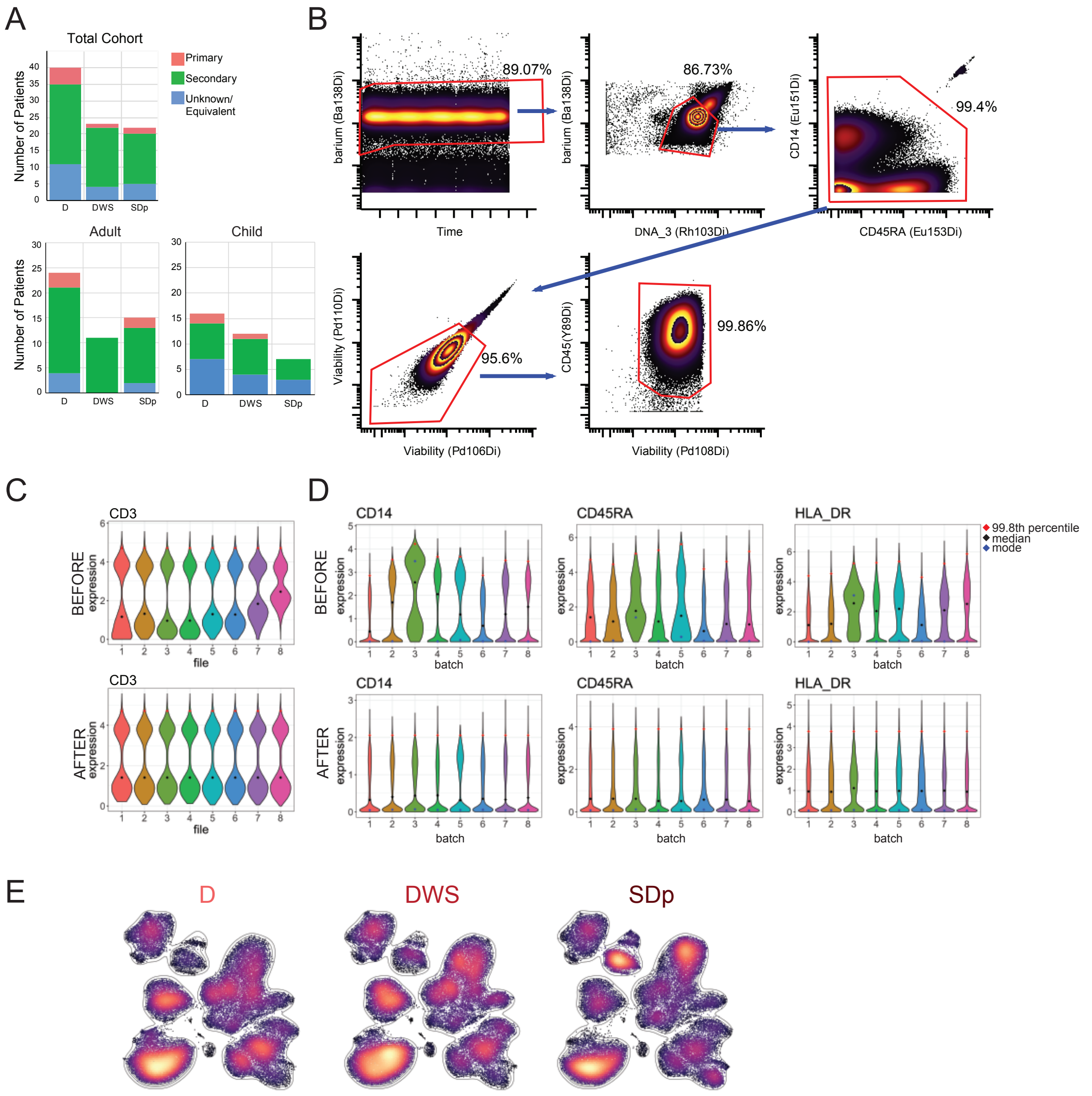

Figure S1: Patient information, gating, normalization, and unbiased segregation of cells by clinical status – related to Figure 1

(A) Composition of cohort by dengue exposure status (colors) and clinical status (columns) in the total cohort and by age category.
(B) Exemplative leukocyte gating strategy
(C) Exemplative correction of CD3 within a batch. Violins represent cell distributions of barcoded files not yet deconvolved that were iteratively collected over the course of a day.
(D) Exemplative batch correction of three protein channels. Violins represent cell distributions from a common control sample run with each batch.
(E) UMAP plots with same coordinates as in Figure 1C, separated by disease status and colored by density.

Figure S2

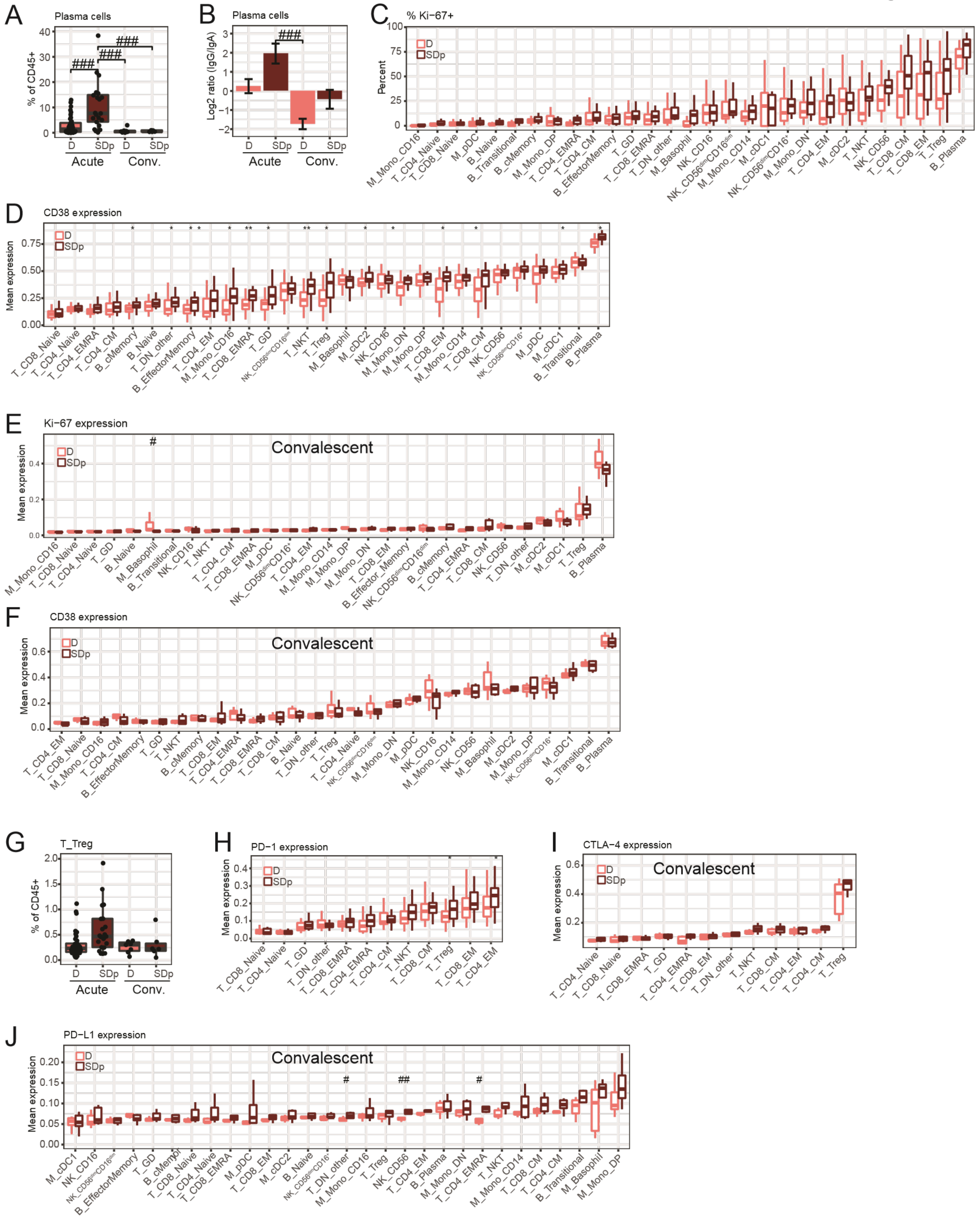

Figure S2. Additional features of immune activation and regulation in acute and convalescent SD and D samples – related to Figure 2

(A, G) Plasma cell and Treg abundance by clinical status and time. Dots represent patients.

(B) Mean $\log_2$ ratio of patient IgG^+ plasma cell abundance to IgA^+ plasma cell abundance by clinical status and time. Error bars represent standard error of mean (SEM).
(C) % Ki-67+ by cell population and clinical status in acute samples.
(D, H) Mean CD38 (D) and PD-1 (H) expression by cell population and clinical status in acute samples.
(E, F, I, J) Mean Ki-67 (E), CD38 (F), CTLA-4 (I), and PD-L1 (J) expression by cell population and clinical status in convalescent samples.
In boxplots, center line signifies median, box signifies interquartile range (IQR) and whiskers signify IQR +/- 1.5*IQR. q<0.05 & |effect|>0.5; ** q<0.01 & |effect|>0.5; *** q<0.005 & |effect|>0.5; # p<0.05 & |effect|>1.0; # p<0.05 & |effect|>1.0; ## p<0.01 & |effect|>1.0; ### p<0.005 & |effect|>1.0 by Wilcoxon rank sum tests. D, dengue; SDp, SD progressors.

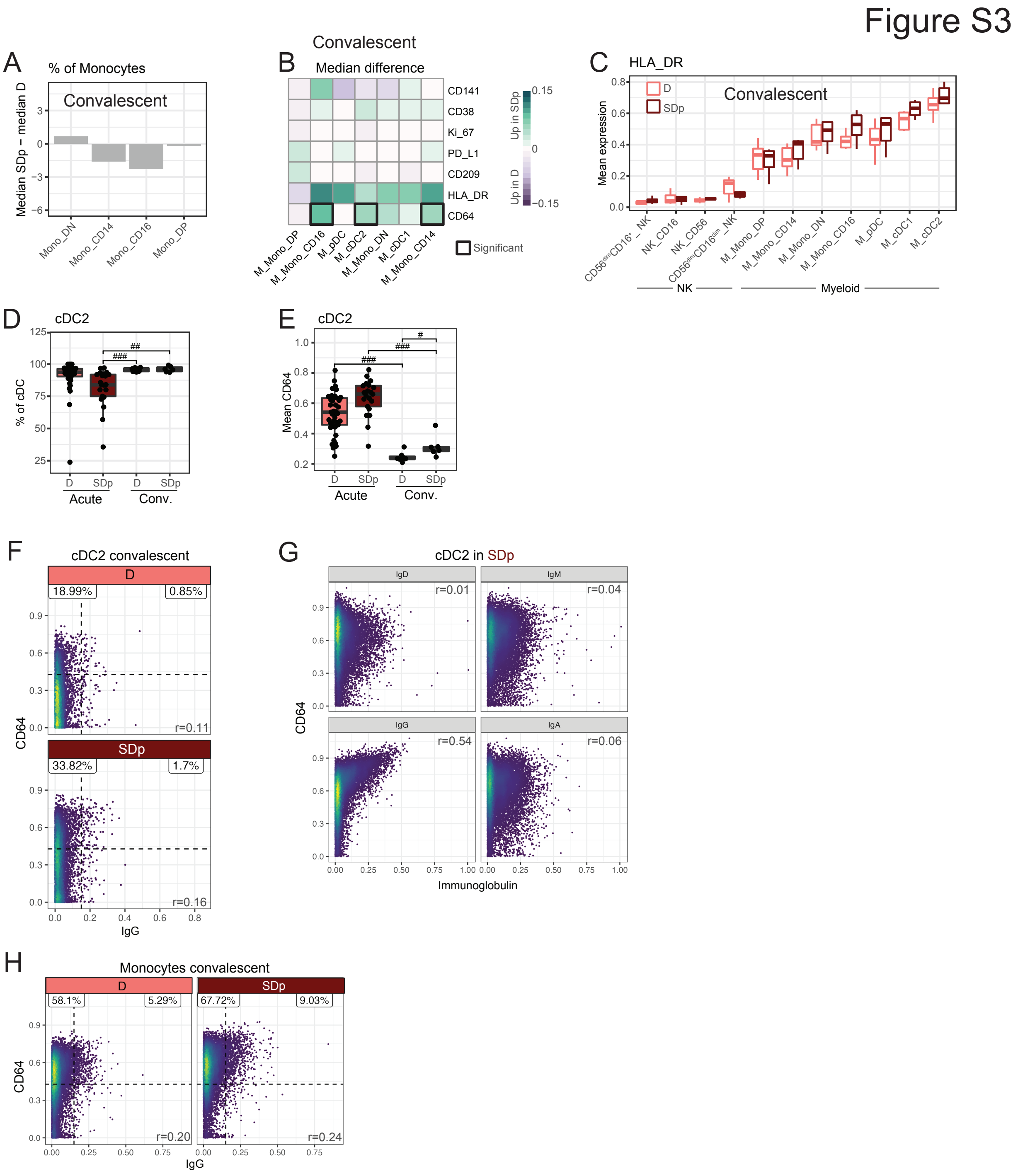

Figure S3: Additional innate immune features of acute and convalescent SDp and D samples – related to Figure 3

(A) Difference in $\log_2$ ratio of median relative abundances of monocyte subtypes (columns) between convalescent SDp and D patients out of total monocytes. Teal bars indicate significance (q<0.05 & |effect|>0.5).

(B) Difference in cohort median of patient mean expression between convalescent SDp and D samples across molecules (rows) in myeloid cell populations (columns). Black boxes indicate significance ($p<0.05$ & $|\text{effect}|>0.5$).
(C) Mean HLA-DR expression in NK and myeloid cell subtypes by clinical status in convalescent samples.
(D, E) Box plots of cDC2 fraction out of total cDCs (D) and mean CD64 expression (E) by clinical status in acute and convalescent samples. Dots represent individual patients.
(F, H) Scatter plots of single cell CD64 expression versus IgG detection in convalescent samples in cDC2s (F) and monocytes (H) by clinical status. Percent proportion of cells in each quadrant, derived from an equal subsampling of cells by clinical status and patient are shown.
(G) Scatter plots of single cell CD64 expression versus immunoglobulin isotypes detection on cDC2s in acute SDp samples. Cells were derived from an equal subsampling of cDC2s by patient.
In boxplots, center line signifies median, box signifies interquartile range (IQR) and whiskers signify IQR +/- 1.5*IQR. Dots in A, C, D, F, and H represent individual patients.
* $q<0.05$ & $|\text{effect}|>0.5$; ** $q<0.01$ & $|\text{effect}|>0.5$; *** $q<0.005$ & $|\text{effect}|>0.5$; # $p<0.05$ & $|\text{effect}|>1.0$; ## $p<0.01$ & $|\text{effect}|>1.0$; ### $p<0.005$ & $|\text{effect}|>1.0$ by Wilcoxon rank sum tests. Q-values represent FDR-corrected p-values. r in F, G, H and I was calculated by Pearson correlation. D, dengue; SDp, SD progressors; conv., convalescent.

Figure S4

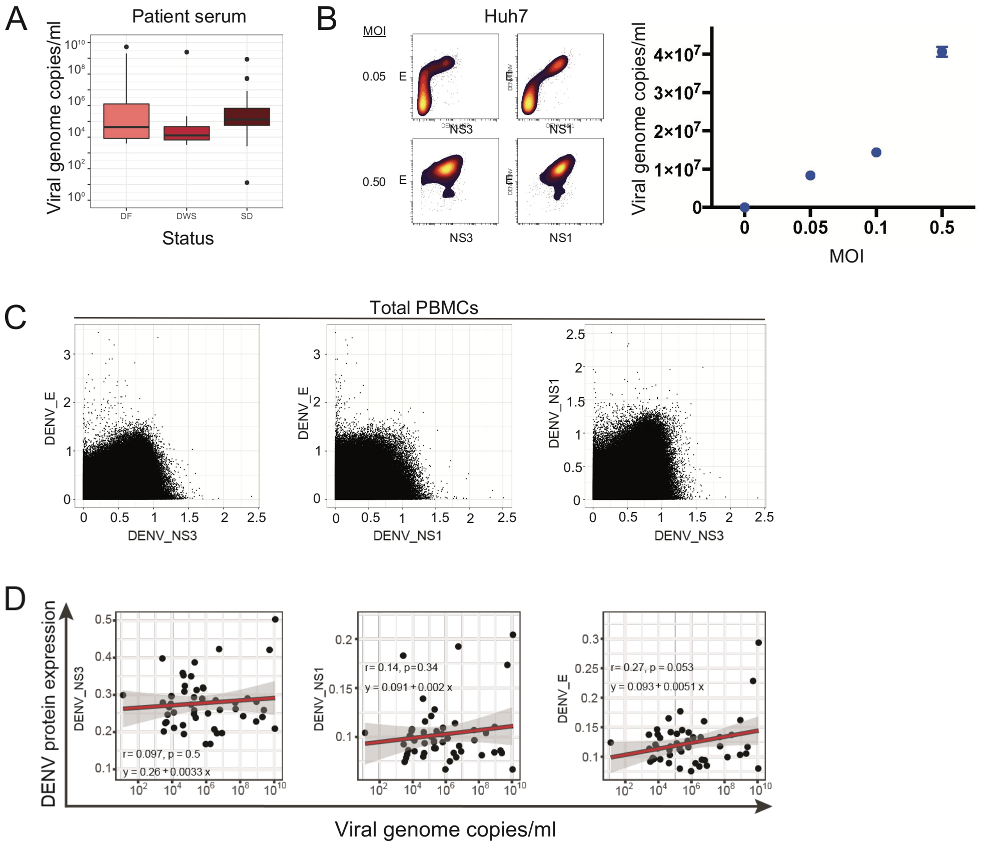

Figure S4: Quantification of DENV viral load and evaluation of DENV target cells via CyTOF

(A) Boxplot depicting level of viremia determined by qRT-PCR by dengue clinical status. In boxplots, center line signifies median, box signifies interquartile range (IQR) and whiskers signify IQR +/- 1.5*IQR.
(B) CyTOF analysis of Huh7 cells infected with DENV-2 at MOIs of 0.05 and 0.5. Left panel: Dot plots of mean signal intensity of DENV E, NS1 and NS3 proteins. Right panel: Viral load measured via qRT-PCR for various MOIs.
(C) Contour plots of single cell DENV E, NS1 and NS3 protein expression in total PBMCs derived from the dengue Colombia cohort.
(D) Scatter plots of mean DENV E, NS1 and NS3 protein expression in PBMCs versus viral load in serum samples with detectable virus by qRT-PCR. Fitting line and 95% CI (shading) were derived by linear regression. Correlation coefficient (r) and p-values were calculated by Pearson correlation.
E, Envelope; NS1, nonstructural 1; NS3, nonstructural 3.

Figure S5

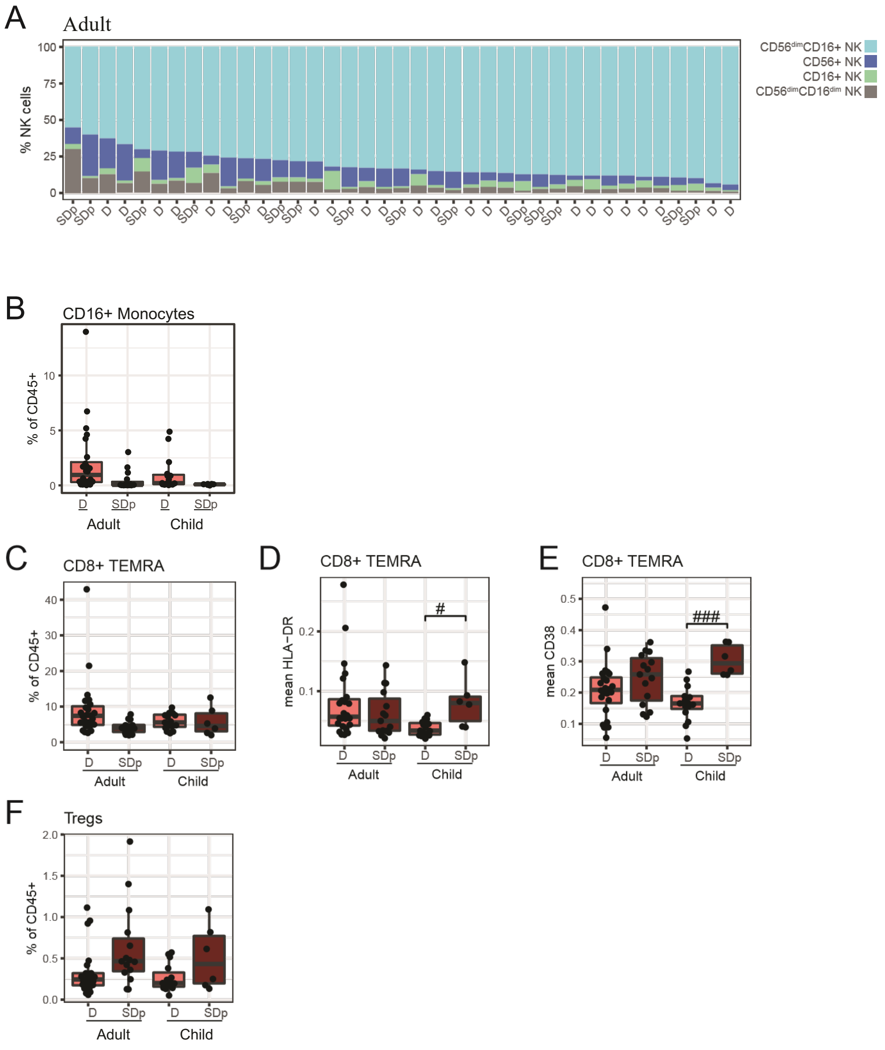

Figure S5: Additional features differentiating SDp children from SDp adults – related to Figure 4

(A) Proportion of NK cell subtypes in adults ordered by $CD56^{dim}CD16^{+}$ NK cell abundance. Columns represent individual patients, labeled by clinical status.

(B, C, F) Box plots of CD16+ monocytes (B) CD8+ TEMRA (C), Treg (F) fractions of CD45+ cells by clinical status and age. Dots represent individual patients.

(D, E) Box plots of mean HLA-DR (D) and CD38 (E) expression in CD8+ TEMRA cells by clinical status and age. Dots represent patients.

In boxplots, center line signifies median, box signifies interquartile range (IQR) and whiskers signify IQR +/- 1.5*IQR. # $p<0.05$ & |effect|>1.0; ## $p<0.01$ & |effect|>1.0; ### $p<0.005$ & |effect|>1.0 by Wilcoxon rank sum tests. D, dengue; SDp, SD progressors.

Figure S6

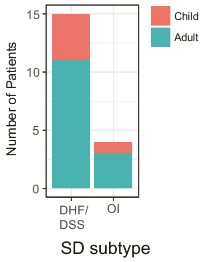

Figure S6: SDp patient distribution by SD subtypes and age – related to Figure 5
Patient composition of SD samples by age (colors) and SD subtype (columns).
DHF/DSS, Dengue hemorrhagic fever/dengue shock syndrome; OI, organ impairment.

Figure S7

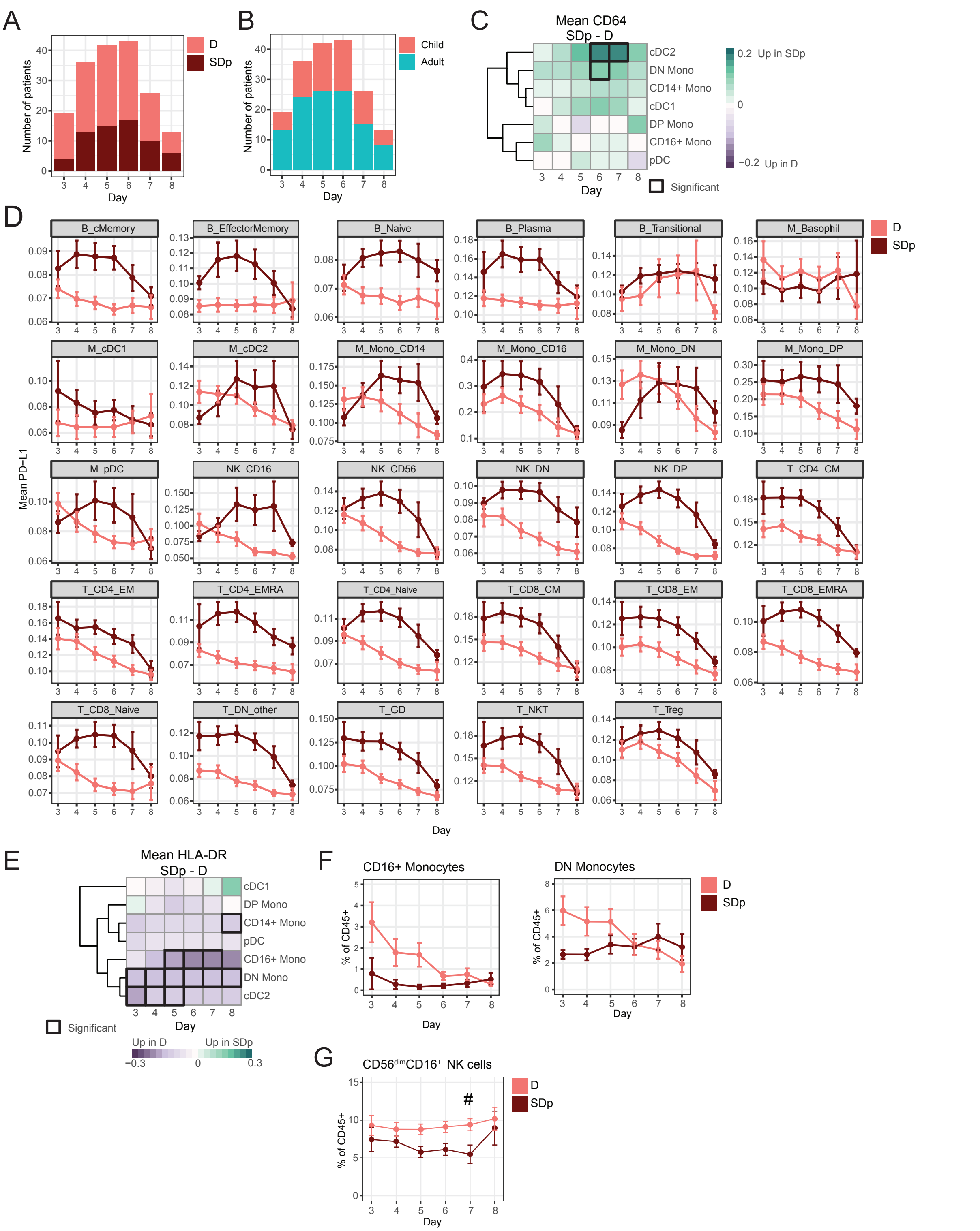

A
Number of patients
Day
D
SDp
B
Child
Adult
C
Mean CD64
SDp - D
cDC2
DN Mono
CD14+ Mono
cDC1
DP Mono
CD16+ Mono
pDC
0.2 Up in SDp
0
−0.2 Up in D
Significant
D
Mean PD−L1
B_cMemory
B_EffectorMemory
B_Naive
B_Plasma
B_Transitional
M_Basophil
M_cDC1
M_cDC2
M_Mono_CD14
M_Mono_CD16
M_Mono_DN
M_Mono_DP
M_pDC
NK_CD16
NK_CD56
NK_DN
NK_DP
T_CD4_CM
T_CD4_EM
T_CD4_EMRA
T_CD4_Naive
T_CD8_CM
T_CD8_EM
T_CD8_EMRA
T_CD8_Naive
T_DN_other
T_GD
T_NKT
T_Treg
E
Mean HLA-DR
SDp - D
Up in D
Up in SDp
−0.3
0.3
F
CD16+ Monocytes
DN Monocytes
% of CD45+
G
CD56dimCD16+ NK cells
#

Figure S7: Additional features of acute infection by time – related to Figure 6
(A) Composition of clinical status (colors) by day.
(B) Composition of age (colors) by day.
(C, E) Difference in cohort mean of patient mean CD64 (D) and HLA-DR (E) expression between SDp and D across cell populations (rows) by day (columns). Black boxes indicate significance ($p<0.05$ & $|effect|>1.0$).
(D) Cohort mean of patient mean PD-L1 expression by day (columns) and clinical status (color), in the indicated cell populations. Each graph is individually scaled.
(F) Cohort mean of $CD16^{+}$ (left) and DN (right) monocyte abundances of CD45+ cells by day (columns) and clinical status (color).
(G) Cohort mean of $CD56^{dim}CD16^{+}$ NK cell abundance of CD45+ cells by day (columns) and clinical status (color).
Error bars represent SEM. D, dengue; SDp, SD progressor; DN, double negative.

Supplementary table captions:

Table S1: Clinical, demographic and laboratory characteristics of patients in the Colombia dengue cohort.
(S1.1) Clinical, demographic and laboratory characteristics of all the patients included in this study.
(S1.2) Clinical characteristics of severe dengue progressors included in this study.
(S1.3) Summary of patient characteristics by disease severity category.
(S1.4) Longitudinal samples analyzed.

Table S2: Effect sizes and p values of pairwise Wilcoxon rank sum tests on cell abundance and protein expression between disease categories in acute samples.

Table S3: Effect sizes and p values of pairwise Wilcoxon rank sum tests on cell abundance and protein expression between acute and convalescent samples.

Table S4: Effect sizes and p values of pairwise Wilcoxon rank sum tests on cell abundance and protein expression between adults and children.

Table S5: Effect sizes and p values of pairwise Wilcoxon rank sum tests on cell abundance and protein expression between severe dengue categories.

Table S6: Effect sizes and p values of pairwise Wilcoxon rank sum tests on cell abundance and protein expression between disease categories on days 3-8 following fever onset.

REFERENCES AND NOTES

1. S. Bhatt, P. W. Gething, O. J. Brady, J. P. Messina, A. W. Farlow, C. L. Moyes, J. M. Drake, J. S. Brownstein, A. G. Hoen, O. Sankoh, M. F. Myers, D. B. George, T. Jaenisch, G. R. W. Wint, C. P. Simmons, T. W. Scott, J. J. Farrar, S. I. Hay, The global distribution and burden of dengue. *Nature* **496**, 504–507 (2013).

2. World Health Organization & UNICEF/UNDP/World Bank/WHO Special Programme for Research and Training in Tropical Diseases, *Handbook for Clinical Management of Dengue* (World Health Organization, 2012); https://apps.who.int/iris/handle/10665/76887.

3. J. D. Stanaway, D. S. Shepard, E. A. Undurraga, Y. A. Halasa, L. E. Coffeng, O. J. Brady, S. I. Hay, N. Bedi, I. M. Bensenor, C. A. Castañeda-Orjuela, T.-W. Chuang, K. B. Gibney, Z. A. Memish, A. Rafay, K. N. Ukwaja, N. Yonemoto, C. J. L. Murray, The global burden of dengue: An analysis from the Global Burden of Disease Study 2013. *Lancet Infect. Dis.* **16**, 712–723 (2016).

4. Z. Zeng, J. Zhan, L. Chen, H. Chen, S. Cheng, Global, regional, and national dengue burden from 1990 to 2017: A systematic analysis based on the global burden of disease study 2017. *EClinicalMedicine* **32**, 100712 2021.

5. S. R. S. Hadinegoro, The revised WHO dengue case classification: Does the system need to be modified? *Paediatr. Int. Child Health.* **32**, 33–38 (2012).

6. F. Narvaez, G. Gutierrez, M. A. Pérez, D. Elizondo, A. Nuñez, A. Balmaseda, E. Harris, Evaluation of the traditional and revised WHO classifications of dengue disease severity. *PLOS Negl. Trop. Dis.* **5**, e1397 (2011).

7. Y. E. Liu, S. Saul, A. M. Rao, M. L. Robinson, O. L. Agudelo Rojas, A. M. Sanz, M. Verghese, D. Solis, M. Sibai, C. H. Huang, M. K. Sahoo, R. M. Gelvez, N. Bueno, M. I. Estupiñan Cardenas, L. A. Villar Centeno, E. M. Rojas Garrido, F. Rosso, M. Donato, B. A. Pinsky, S. Einav, P. Khatri, An 8-gene machine learning model improves clinical prediction of severe dengue progression. *Genome Med.* **14**, 33 (2022).

8. M. Robinson, T. E. Sweeney, R. Barouch-Bentov, M. K. Sahoo, L. Kalesinskas, F. Vallania, A. M. Sanz, E. Ortiz-Lasso, L. L. Albornoz, F. Rosso, J. G. Montoya, B. A. Pinsky, P. Khatri, S. Einav, A 20-gene set predictive of progression to severe dengue. *Cell Rep.* **26**, 1104–1111.e4 (2019).

9. H. Tissera, A. P. S. Rathore, W. Y. Leong, B. L. Pike, T. E. Warkentien, F. S. Farouk, A. Syenina, E. Eong Ooi, D. J. Gubler, A. Wilder-Smith, A. L. St. John, Chymase level is a predictive biomarker of dengue hemorrhagic fever in pediatric and adult patients. *J. Infect. Dis.* **216**, 1112–1121 (2017).

10. F. Zanini, M. L. Robinson, D. Croote, M. K. Sahoo, A. M. Sanz, E. Ortiz-Lasso, L. L. Albornoz, F. Rosso, J. G. Montoya, L. Goo, B. A. Pinsky, S. R. Quake, S. Einav, Virus-inclusive single-cell RNA sequencing reveals the molecular signature of progression to severe dengue. *Proc. Natl. Acad. Sci. U.S.A.* **115**, E12363–E12369 (2018).

11. S. Halstead, E. O'Rourke, Dengue viruses and mononuclear phagocytes. I. Infection enhancement by non-neutralizing antibody. *J. Exp. Med.* **146**, 201–217 (1977).

12. S. B. Halstead, E. J. O'rourke, Antibody-enhanced dengue virus infection in primate leukocytes. *Nature* **265**, 739–741 (1977).

13. L. C. Katzelnick, L. Gresh, M. E. Halloran, J. C. Mercado, G. Kuan, A. Gordon, A. Balmaseda, E. Harris, Antibody-dependent enhancement of severe dengue disease in humans. *Science* **358**, 929–932 (2017).

14. M. Kwissa, H. I. Nakaya, N. Onlamoon, J. Wrammert, F. Villinger, G. C. Perng, S. Yoksan, K. Pattanapanyasat, K. Chokephaibulkit, R. Ahmed, B. Pulendran, Dengue virus infection induces expansion of a $CD14^{+}CD16^{+}$ monocyte population that stimulates plasmablast differentiation. *Cell Host Microbe* **16**, 115–127 (2014).

15. E. J. M. Nascimento, E. D. Hottz, T. M. Garcia-Bates, F. Bozza, E. T. A. Marques, S. M. Barratt-Boyes, Emerging concepts in dengue pathogenesis: Interplay between plasmablasts, platelets, and complement in triggering vasculopathy. *Crit. Rev. Immunol.* **34**, 227–240 (2014).

16. J. Mongkolsapaya, T. Duangchinda, W. Dejnirattisai, S. Vasanawathana, P. Avirutnan, A. Jairungsri, N. Khemnu, N. Tangthaworhchaikul, P. Chotiyarnwong, K. Sae-Jang, M. Koch, Y. Jones, A.

McMichael, X. Xu, P. Malasit, G. Screaton, T cell responses in dengue hemorrhagic fever: Are cross-reactive T cells suboptimal? *J. Immunol.* **176**, 3821–3829 (2006).

17. S.-T. Chen, Y.-L. Lin, M.-T. Huang, M.-F. Wu, S.-C. Cheng, H.-Y. Lei, C.-K. Lee, T.-W. Chiou, C.-H. Wong, S.-L. Hsieh, CLEC5A is critical for dengue-virus-induced lethal disease. *Nature* **453**, 672–676 (2008).

18. W.-K. Wang, H.-L. Chen, C.-F. Yang, S.-C. Hsieh, C.-C. Juan, S.-M. Chang, C.-C. Yu, L.-H. Lin, J.-H. Huang, C.-C. King, Slower rates of clearance of viral load and virus-containing immune complexes in patients with dengue hemorrhagic fever. *Clin. Infect. Dis.* **43**, 1023–1030 (2006).

19. D. W. Vaughn, S. Green, S. Kalayanarooj, B. L. Innis, S. Nimmannitya, S. Suntayakorn, T. P. Endy, B. Raengsakulrach, A. L. Rothman, F. A. Ennis, A. Nisalak, Dengue viremia titer, antibody response pattern, and virus serotype correlate with disease severity. *J. Infect. Dis.* **181**, 2–9 (2000).

20. M. G. Guzmán, G. Kouri, J. Bravo, L. Valdes, S. Vazquez, S. B. Halstead, Effect of age on outcome of secondary dengue 2 infections. *Int. J. Infect. Dis.* **6**, 118–124 (2002).

21. O. Wichmann, S. Hongsiriwon, C. Bowonwatanuwong, K. Chotivanich, Y. Sukthana, S. Pukrittayakamee, Risk factors and clinical features associated with severe dengue infection in adults and children during the 2001 epidemic in Chonburi, Thailand, *Trop. Med. Int. Health* **9**, 1022–1029 (2004).

22. S. Sangkaew, D. Ming, A. Boonyasiri, K. Honeyford, S. Kalayanarooj, S. Yacoub, I. Dorigatti, A. Holmes, Risk predictors of progression to severe disease during the febrile phase of dengue: A systematic review and meta-analysis. *Lancet Infect. Dis.* **21**, 1014–1026 (2021).

23. A. Karunakaran, W. M. Ilyas, S. F. Sheen, N. K. Jose, Z. T. Nujum, Risk factors of mortality among dengue patients admitted to a tertiary care setting in Kerala, India. *J. Infect. Public Health* **7**, 114–120 (2014).

24. E. Becht, L. McInnes, J. Healy, C.-A. Dutertre, I. W. H. Kwok, L. G. Ng, F. Ginhoux, E. W. Newell, Dimensionality reduction for visualizing single-cell data using UMAP. *Nat. Biotechnol.* **37**, 38–44 (2019).

25. M. Amouzgar, D. R. Glass, R. Baskar, I. Averbukh, S. C. Kimmey, A. G. Tsai, F. J. Hartmann, S. C. Bendall, Supervised dimensionality reduction for exploration of single-cell data by HSS-LDA. *Patterns (N Y)* **3**, 100536 (2022).

26. L. A. Callender, E. C. Carroll, R. W. J. Beal, E. S. Chambers, S. Nourshargh, A. N. Akbar, S. M. Henson, Human $CD8^+$ EMRA T cells display a senescence-associated secretory phenotype regulated by p38 MAPK. *Aging Cell* **17**, e12675 (2018).

27. M. Collin, V. Bigley, Human dendritic cell subsets: An update. *Immunology* **154**, 3–20 (2018).

28. M. Gilliet, W. Cao, Y.-J. Liu, Plasmacytoid dendritic cells: Sensing nucleic acids in viral infection and autoimmune diseases. *Nat. Rev. Immunol.* **8**, 594–606 (2008).

29. J. K. Krishnaswamy, U. Gowthaman, B. Zhang, J. Mattsson, L. Szeponik, D. Liu, R. Wu, T. White, S. Calabro, L. Xu, M. A. Collet, M. Yurieva, S. Alsén, P. Fogelstrand, A. Walter, W. R. Heath, S. N. Mueller, U. Yrlid, A. Williams, S. C. Eisenbarth, Migratory $CD11b^+$ conventional dendritic cells induce T follicular helper cell-dependent antibody responses. *Sci. Immunol.* **2**, eaam9169 (2017).

30. S. Bournazos, A. Gupta, J. V. Ravetch, The role of IgG Fc receptors in antibody-dependent enhancement. *Nat. Rev. Immunol.* **20**, 633–643 (2020).

31. K. A. Hogan, C. C. S. Chini, E. N. Chini, The multi-faceted ecto-enzyme CD38: Roles in immunomodulation, cancer, aging, and metabolic diseases. *Front. Immunol.* **10**, 1187 (2019).

32. E. I. Buchbinder, A. Desai, CTLA-4 and PD-1 pathways. *Am. J. Clin. Oncol.* **39**, 98–106 (2016).

33. X. Feng, L. Zhang, C. Acharya, G. An, K. Wen, L. Qiu, N. Munshi, Y.-T. Tai, K. C. Anderson, Targeting CD38 suppresses induction and function of T regulatory cells to mitigate immunosuppression in multiple myeloma. *Clin. Cancer Res.* **23**, 4290–4300 (2017).

34. Y.-H. Li, C.-H. Kuo, G.-Y. Shi, H.-L. Wu, The role of thrombomodulin lectin-like domain in inflammation. *J. Biomed. Sci.* **19**, 34 (2012).

35. B. Tassaneetrithep, T. H. Burgess, A. Granelli-Piperno, C. Trumpfheller, J. Finke, W. Sun, M. A. Eller, K. Pattanapanyasat, S. Sarasombath, D. L. Birx, R. M. Steinman, S. Schlesinger, M. A. Marovich, DC-SIGN (CD209) mediates dengue virus infection of human dendritic cells. *J. Exp. Med.* **197**, 823–829 (2003).

36. S. A. Erokhina, M. A. Streltsova, L. M. Kanevskiy, W. G. Telford, A. M. Sapozhnikov, E. I. Kovalenko, HLA-DR^+ NK cells are mostly characterized by less mature phenotype and high functional activity. *Immunol. Cell Biol.* **96**, 212–228 (2018).

37. S. A. Kust, M. A. Streltsova, A. V. Panteleev, N. L. Karpina, I. V. Lyadova, A. M. Sapozhnikov, E. I. Kovalenko, HLA-DR-positive NK cells expand in response to mycobacterium tuberculosis antigens and mediate mycobacteria-induced T cell activation. *Front. Immunol.* **12**, 662128 (2021).

38. D. G. Schatz, Y. Ji, Recombination centres and the orchestration of V(D)J recombination. *Nat. Rev. Immunol.* **11**, 251–263 (2011).

39. P. Bruhns, B. Iannascoli, P. England, D. A. Mancardi, N. Fernandez, S. Jorieux, M. Daëron, Specificity and affinity of human Fcγ receptors and their polymorphic variants for human IgG subclasses. *Blood* **113**, 3716–3725 (2009).

40. T. T. Wang, J. Sewatanon, M. J. Memoli, J. Wrammert, S. Bournazos, S. K. Bhaumik, B. A. Pinsky, K. Chokephaibulkit, N. Onlamoon, K. Pattanapanyasat, J. K. Taubenberger, R. Ahmed, J. V. Ravetch, IgG antibodies to dengue enhanced for FcγRIIIA binding determine disease severity. *Science* **355**, 395–398 (2017).

41. S. J. Balsitis, J. Coloma, G. Castro, A. Alava, D. Flores, J. H. McKerrow, P. R. Beatty, E. Harris, Tropism of dengue virus in mice and humans defined by viral nonstructural protein 3-specific immunostaining. *Am. J. Trop. Med. Hyg.* **80**, 416–424 (2009).

42. S. N. Hammond, A. Balmaseda, L. Pérez, Y. Tellez, S. I. Saborío, J. C. Mercado, E. Videa, Y. Rodriguez, M. A. Pérez, R. Cuadra, S. Solano, J. Rocha, W. Idiaquez, A. Gonzalez, E. Harris, Differences in dengue severity in infants, children, and adults in a 3-year hospital-based study in Nicaragua. *Am. J. Trop. Med. Hyg.* **73**, 1063–1070 (2005).

43. K. Srpan, A. Ambrose, A. Karampatzakis, M. Saeed, A. N. R. Cartwright, K. Guldevall, G. D. S. C. De Matos, B. Önfelt, D. M. Davis, Shedding of CD16 disassembles the NK cell immune synapse and boosts serial engagement of target cells. *J. Cell Biol.* **217**, 3267–3283 (2018).

44. G. Peruzzi, L. Femnou, A. Gil-Krzewska, F. Borrego, J. Weck, K. Krzewski, J. E. Coligan, Membrane-type 6 matrix metalloproteinase regulates the activation-induced downmodulation of CD16 in human primary NK cells. *J. Immunol.* **191**, 1883–1894 (2013).

45. F. Borrego, M. J. Robertson, J. Ritz, J. Peña, R. Solana, CD69 is a stimulatory receptor for natural killer cell and its cytotoxic effect is blocked by CD94 inhibitory receptor. *Immunology* **97**, 159–165 (1999).

46. M. C. Glass, D. R. Glass, J.-P. Oliveria, B. Mbiribindi, C. O. Esquivel, S. M. Krams, S. C. Bendall, O. M. Martinez, Human IL-10-producing B cells have diverse states that are induced from multiple B cell subsets. *Cell Rep.* **39**, 110728 (2022).

47. Y. Zhao, M. Amodio, B. V. Wyk, B. Gerritsen, M. M. Kumar, D. van Dijk, K. Moon, X. Wang, A. Malawista, M. M. Richards, M. E. Cahill, A. Desai, J. Sivadasan, M. M. Venkataswamy, V. Ravi, E. Fikrig, P. Kumar, S. H. Kleinstein, S. Krishnaswamy, R. R. Montgomery, Single cell immune profiling of dengue virus patients reveals intact immune responses to Zika virus with enrichment of innate immune signatures. *PLOS Negl. Trop. Dis.* **14**, e0008112 (2020).

48. R. Fenutria, K. Maringer, U. Potla, D. Bernal-Rubio, M. J. Evans, E. Harris, A. H. Rahman, A. Fernandez-Sesma, I. Ramos, CyTOF profiling of Zika and dengue virus-infected human peripheral blood mononuclear cells identifies phenotypic signatures of monotype subsets and upregulation of the interferon-inducible protein CD169. *mSphere* **6**, e0050521 (2021).

49. A. Rouers, M. H. Y. Chng, B. Lee, M. P. Rajapakse, K. Kaur, Y. X. Toh, D. Sathiakumar, T. Loy, T.-L. Thein, V. W. X. Lim, A. Singhal, T. W. Yeo, Y.-S. Leo, K. A. Vora, D. Casimiro, B. Lim, L. Tucker-Kellogg, L. Rivino, E. W. Newell, K. Fink, Immune cell phenotypes associated with disease severity and long-term neutralizing antibody titers after natural dengue virus infection. *Cell Rep. Med.* **2**, 100278 (2021).

50. R. Appanna, S. Kg, M. H. Xu, Y.-X. Toh, S. Velumani, D. Carbajo, C. Y. Lee, R. Zuest, T. Balakrishnan, W. Xu, B. Lee, M. Poidinger, F. Zolezzi, Y. S. Leo, T. L. Thein, C.-I. Wang, K. Fink, Plasmablasts during acute dengue infection represent a small subset of a broader virus-specific memory B cell pool. *EBioMedicine* **12**, 178–188 (2016).

51. A. L. Rothman, Immunity to dengue virus: A tale of original antigenic sin and tropical cytokine storms. *Nat. Rev. Immunol.* **11**, 532–543 (2011).

52. K. Buscher, P. Marcovecchio, C. C. Hedrick, K. Ley, Patrolling mechanics of non-classical monocytes in vascular inflammation. *Front. Cardiovasc. Med.* **4**, 80 (2017).

53. S. A. Marsh, H. M. Arthur, I. Spyridopoulos, The secret life of nonclassical monocytes. *Cytometry A* **91**, 1055–1058 (2017).

54. D. Maheshwari, K. Saini, P. Singh, M. Singla, K. Nayak, C. Aggarwal, Y. M. Chawla, P. Bajpai, M. Kaur, S. Gunisetty, C. S. Eberhardt, R. Nyodu, K. Moore, M. S. Suthar, G. R. Medigeshi, E. Anderson, R. Lodha, S. K. Kabra, R. Ahmed, A. Chandele, K. Murali-Krishna, Contrasting behavior between the three human monocyte subsets in dengue pathophysiology. *iScience* **25**, 104384 (2022).

55. T. Chawla, K. R. Chan, S. L. Zhang, H. C. Tan, A. P. C. Lim, B. J. Hanson, E. E. Ooi, Dengue virus neutralization in cells expressing Fc gamma receptors. *PLOS ONE* **8**, e65231 (2013).

56. W. W. S. I. Rodrigo, O. K. T. Block, C. Lane, S. Sukupolvi-Petty, A. P. Goncalvez, S. Johnson, M. S. Diamond, C.-J. Lai, R. C. Rose, X. Jin, J. J. Schlesinger, Dengue virus neutralization is modulated by IgG antibody subclass and Fcγ receptor subtype. *Virology* **394**, 175–182 (2009).

57. A. Silvin, C. I. Yu, X. Lahaye, F. Imperatore, J.-B. Brault, S. Cardinaud, C. Becker, W.-H. Kwan, C. Conrad, M. Maurin, C. Goudot, S. Marques-Ladeira, Y. Wang, V. Pascual, E. Anguiano, R. A. Albrecht, M. Iannacone, A. García-Sastre, B. Goud, M. Dalod, A. Moris, M. Merad, A. K. Palucka, N. Manel, Constitutive resistance to viral infection in human $CD141^+$ dendritic cells. *Sci. Immunol.* **2**, eaai8071 (2017).

58. C.-C. Chu, N. Ali, P. Karagiannis, P. Di Meglio, A. Skowera, L. Napolitano, G. Barinaga, K. Grys, E. Sharif-Paghaleh, S. N. Karagiannis, M. Peakman, G. Lombardi, F. O. Nestle, Resident CD141

(BDCA3)$^{+}$ dendritic cells in human skin produce IL-10 and induce regulatory T cells that suppress skin inflammation. *J. Exp. Med.* **209**, 935–945 (2012).

59. N. van Leeuwen-Kerkhoff, T. M. Westers, P. J. Poddighe, T. D. de Gruijl, S. Kordasti, A. A. van de Loosdrecht, Thrombomodulin-expressing monocytes are associated with low-risk features in myelodysplastic syndromes and dampen excessive immune activation. *Haematologica* **105**, 961–971 (2020).

60. J. P. Ren, J. Zhao, J. Dai, J. W. D. Griffin, L. Wang, X. Y. Wu, Z. D. Morrison, G. Y. Li, M. El Gazzar, S. B. Ning, J. P. Moorman, Z. Q. Yao, Hepatitis C virus-induced myeloid-derived suppressor cells regulate T-cell differentiation and function via the signal transducer and activator of transcription 3 pathway. *Immunology* **148**, 377–386 (2016).

61. L. Wang, J. Zhao, J. P. Ren, X. Y. Wu, Z. D. Morrison, M. A. Elgazzar, S. B. Ning, J. P. Moorman, Z. Q. Yao, Expansion of myeloid-derived suppressor cells promotes differentiation of regulatory T cells in HIV-1^{+} individuals. *AIDS* **30**, 1521–1531 (2016).

62. K. Lühn, C. P. Simmons, E. Moran, N. T. P. Dung, T. N. B. Chau, N. T. H. Quyen, L. T. T. Thao, T. Van Ngoc, N. M. Dung, B. Wills, J. Farrar, A. J. McMichael, T. Dong, S. Rowland-Jones, Increased frequencies of CD4^{+}CD25^{high} regulatory T cells in acute dengue infection. *J. Exp. Med.* **204**, 979–985 (2007).

63. H. E. Jayaratne, D. Wijeratne, S. Fernando, A. Kamaladasa, L. Gomes, A. Wijewickrama, G. S. Ogg, G. N. Malavige, Regulatory T-cells in acute dengue viral infection. *Immunology* **154**, 89–97 (2018).

64. P. Pandiyan, L. Zheng, S. Ishihara, J. Reed, M. J. Lenardo, CD4^{+}CD25^{+}Foxp3^{+} regulatory T cells induce cytokine deprivation-mediated apoptosis of effector CD4^{+} T cells. *Nat. Immunol.* **8**, 1353–1362 (2007).

65. W. Pierson, B. Cauwe, A. Policheni, S. M. Schlenner, D. Franckaert, J. Berges, S. Humblet-Baron, S. Schonefeldt, M. J. Herold, D. Hildeman, A. Strasser, P. Bouillet, L.-F. Lu, P. Matthys, A. A. Freitas, R. J. Luther, C. T. Weaver, J. Dooley, D. H. D. Gray, A. Liston, Anti-apoptotic Mcl-1 is critical for the survival and niche-filling capacity of Foxp3^{+} regulatory T cells. *Nat. Immunol.* **14**, 959–965 (2013).

66. P. Ruibal, L. Oestereich, A. Lüdtke, B. Becker-Ziaja, D. M. Wozniak, R. Kerber, M. Korva, M. Cabeza-Cabrerizo, J. A. Bore, F. R. Koundouno, S. Duraffour, R. Weller, A. Thorenz, E. Cimini, *D. Viola*, C. Agrati, J. Repits, B. Afrough, L. A. Cowley, D. Ngabo, J. Hinzmann, M. Mertens, I. Vitoriano, C. H. Logue, J. P. Boettcher, E. Pallasch, A. Sachse, A. Bah, K. Nitzsche, E. Kuisma, J. Michel, T. Holm, E.-G. Zekeng, I. García-Dorival, R. Wölfel, K. Stoecker, E. Fleischmann, T. Strecker, A. Di Caro, T. Avšič-Županc, A. Kurth, S. Meschi, S. Mély, E. Newman, A. Bocquin, Z. Kis, A. Kelterbaum, P. Molkenthin, F. Carletti, J. Portmann, S. Wolff, C. Castilletti, G. Schudt, A. Fizet, L. J. Ottowell, E. Herker, T. Jacobs, B. Kretschmer, E. Severi, N. Ouedraogo, M. Lago, A. Negredo, L. Franco, P. Anda, S. Schmiedel, B. Kreuels, D. Wichmann, M. M. Addo, A. W. Lohse, H. De Clerck, C. Nanclares, S. Jonckheere, M. Van Herp, A. Sprecher, G. Xiaojiang, M. Carrington, O. Miranda, C. M. Castro, M. Gabriel, P. Drury, P. Formenty, B. Diallo, L. Koivogui, N. Magassouba, M. W. Carroll, S. Günther, C. Muñoz-Fontela, Unique human immune signature of Ebola virus disease in Guinea. *Nature* **533**, 100–104 (2016).

67. R.-F. Chen, L. Wang, J.-T. Cheng, H. Chuang, J.-C. Chang, J.-W. Liu, I.-C. Lin, K. D. Yang, Combination of CTLA-4 and TGFβ1 gene polymorphisms associated with dengue hemorrhagic fever and virus load in a dengue-2 outbreak. *Clin. Immunol.* **131**, 404–409 (2009).

68. E. Simon-Lorière, V. Duong, A. Tawfik, S. Ung, S. Ly, I. Casadémont, M. Prot, N. Courtejoie, K. Bleakley, P. Buchy, A. Tarantola, P. Dussart, T. Cantaert, A. Sakuntabhai, Increased adaptive immune responses and proper feedback regulation protect against clinical dengue. *Sci. Transl. Med.* **9**, eaal5088 (2017).

69. J.-M. Sung, C.-K. Lee, B. A. Wu-Hsieh, Intrahepatic infiltrating NK and CD8 T cells cause liver cell death in different phases of dengue virus infection. *PLOS ONE* **7**, e46292 (2012).

70. D. Ricklin, J. D. Lambris, Complement in immune and inflammatory disorders: Pathophysiological mechanisms. *J. Immunol.* **190**, 3831–3838 (2013).

71. M. Xu, V. Hadinoto, R. Appanna, K. Joensson, Y. X. Toh, T. Balakrishnan, S. H. Ong, L. Warter, Y. S. Leo, C.-I. Wang, K. Fink, Plasmablasts generated during repeated dengue infection are virus glycoprotein-specific and bind to multiple virus serotypes. *J. Immunol.* **189**, 5877–5885 (2012).

72. S. Green, S. Pichyangkul, D. W. Vaughn, S. Kalayanarooj, S. Nimmannitya, A. Nisalak, I. Kurane, A. L. Rothman, F. A. Ennis, Early CD69 expression on peripheral blood lymphocytes from children with dengue hemorrhagic fever. *J. Infect. Dis.* **180**, 1429–1435 (1999).

73. R. Romee, B. Foley, T. Lenvik, Y. Wang, B. Zhang, D. Ankarlo, X. Luo, S. Cooley, M. Verneris, B. Walcheck, J. Miller, NK cell CD16 surface expression and function is regulated by a disintegrin and metalloprotease-17 (ADAM17). *Blood* **121**, 3599–3608 (2013).

74. V. Béziat, D. Duffy, S. N. Quoc, M. Le Garff-Tavernier, J. Decocq, B. Combadière, P. Debré, V. Vieillard, $CD56^{bright}CD16^{+}$ NK cells: A functional intermediate stage of NK cell differentiation. *J. Immunol.* **186**, 6753–6761 (2011).

75. R. Castriconi, P. Carrega, A. Dondero, F. Bellora, B. Casu, S. Regis, G. Ferlazzo, C. Bottino, Molecular mechanisms directing migration and retention of natural killer cells in human tissues. *Front. Immunol.* **9**, 2324 (2018).

76. C. L. Zimmer, M. Cornillet, C. Solà-Riera, K.-W. Cheung, M. A. Ivarsson, M. Q. Lim, N. Marquardt, Y.-S. Leo, D. C. Lye, J. Klingström, P. A. MacAry, H.-G. Ljunggren, L. Rivino, N. K. Björkström, NK cells are activated and primed for skin-homing during acute dengue virus infection in humans. *Nat. Commun.* **10**, 3897 (2019).

77. C. R. Machado, E. S. Machado, R. D. Rohloff, M. Azevedo, D. P. Campos, R. B. de Oliveira, P. Brasil, Is pregnancy associated with severe dengue? A review of data from the Rio de Janeiro surveillance information system. *PLOS Negl. Trop. Dis.* **7**, e2217 (2013).

78. E. M. Gainor, E. Harris, A. D. LaBeaud, Uncovering the burden of dengue in Africa: Considerations on magnitude, misdiagnosis, and sncestry. *Viruses* **14**, 233 (2022).

79. B. D. Kirkpatrick, S. S. Whitehead, K. K. Pierce, C. M. Tibery, P. L. Grier, N. A. Hynes, C. J. Larsson, B. P. Sabundayo, K. R. Talaat, A. Janiak, M. P. Carmolli, C. J. Luke, S. A. Diehl, A. P. Durbin, The live attenuated dengue vaccine TV003 elicits complete protection against dengue in a human challenge model. *Sci. Transl. Med.* **8**, 330ra36 (2016).

80. A. T. Waickman, K. Newell, J. Q. Lu, H. Fang, M. Waldran, C. Gebo, J. R. Currier, H. Friberg, R. G. Jarman, M. D. Klick, L. A. Ware, T. P. Endy, S. J. Thomas, Virologic, clinical, and immunological characteristics of a dengue virus 3 human challenge model. medrxiv 2022.10.24.22281454 (2022). https://doi.org/10.1101/2022.10.24.22281454.

81. S. Bournazos, H. T. M. Vo, V. Duong, H. Auerswald, S. Ly, A. Sakuntabhai, P. Dussart, T. Cantaert, J. V. Ravetch, Antibody fucosylation predicts disease severity in secondary dengue infection. *Science* **372**, 1102–1105 (2021).

82. A. Mauriello, C. Manolio, B. Cavalluzzo, A. Avallone, M. Borrelli, A. Morabito, E. Iovine, A. Chambery, R. Russo, M. L. Tornesello, F. M. Buonaguro, M. Tagliamonte, L. Buonaguro, Immunological effects of adjuvants in subsets of antigen presenting cells of cancer patients undergoing chemotherapy. *J. Transl. Med.* **18**, 34 (2020).

83. M. N. Wykes, S. R. Lewin, Immune checkpoint blockade in infectious diseases. *Nat. Rev. Immunol.* **18**, 91–104 (2018).

84. S. Urbani, B. Amadei, D. Tola, M. Massari, S. Schivazappa, G. Missale, C. Ferrari, PD-1 expression in acute hepatitis C virus (HCV) infection is associated with HCV-specific CD8 exhaustion. *J. Virol.* **80**, 11398–11403 (2006).

85. B. McNally, F. Ye, M. Willette, E. Flaño, Local blockade of epithelial PDL-1 in the airways enhances T cell function and viral clearance during influenza virus infection. *J. Virol.* **87**, 12916–12924 (2013).

86. J. J. Waggoner, L. Gresh, A. Mohamed-Hadley, G. Ballesteros, M. J. V. Davila, Y. Tellez, M. K. Sahoo, A. Balmaseda, E. Harris, B. A. Pinsky, Single-reaction multiplex reverse transcription PCR for detection of Zika, chikungunya, and dengue viruses. *Emerg. Infect. Dis.* **22**, 1295–1297 (2016).

87. J. J. Waggoner, J. Abeynayake, M. K. Sahoo, L. Gresh, Y. Tellez, K. Gonzalez, G. Ballesteros, A. M. Pierro, P. Gaibani, F. P. Guo, V. Sambri, A. Balmaseda, K. Karunaratne, E. Harris, B. A. Pinsky, Single-reaction, multiplex, real-time RT-PCR for the detection, quantitation, and serotyping of dengue viruses. *PLOS Negl. Trop. Dis.* **7**, e2116 (2013).

88. B. Zhang, B. A. Pinsky, J. S. Ananta, S. Zhao, S. Arulkumar, H. Wan, M. K. Sahoo, J. Abeynayake, J. J. Waggoner, C. Hopes, M. Tang, H. Dai, Diagnosis of Zika virus infection on a nanotechnology platform. *Nat. Med.* **23**, 548–550 (2017).

89. F. J. Hartmann, J. Babdor, P. F. Gherardini, E.-A. D. Amir, K. Jones, B. Sahaf, D. M. Marquez, P. Krutzik, E. O'Donnell, N. Sigal, H. T. Maecker, E. Meyer, M. H. Spitzer, S. C. Bendall, Comprehensive immune monitoring of clinical trials to advance human immunotherapy. *Cell Rep.* **28**, 819–831.e4 (2019).

90. F. J. Hartmann, E. F. Simonds, S. C. Bendall, A universal live cell barcoding-platform for multiplexed human single cell analysis. *Sci. Rep.* **8**, 10770 (2018).

91. B. Sahaf, A. Rahman, H. T. Maecker, S. Bendall, High-parameter immune profiling with CyTOF. *Methods Mol. Biol.* **2055**, 351–368 (2020).

92. R. Finck, E. F. Simonds, A. Jager, S. Krishnaswamy, K. Sachs, W. Fantl, D. Pe'er, G. P. Nolan, S. C. Bendall, Normalization of mass cytometry data with bead standards. *Cytometry A* **83**, 483–494 (2013).

93. A. G. Tsai, D. R. Glass, M. Juntilla, F. J. Hartmann, J. S. Oak, S. Fernandez-Pol, R. S. Ohgami, S. C. Bendall, Multiplexed single-cell morphometry for hematopathology diagnostics. *Nat. Med.* **26**, 408–417 (2020).

94. S. Chevrier, H. L. Crowell, V. R. T. Zanotelli, S. Engler, M. D. Robinson, B. Bodenmiller, Compensation of signal spillover in suspension and imaging mass cytometry. *Cell Syst.* **6**, 612–620.e5 (2018).

95. D. R. Glass, A. G. Tsai, J. P. Oliveria, F. J. Hartmann, S. C. Kimmey, A. A. Calderon, L. Borges, M. C. Glass, L. E. Wagar, M. M. Davis, S. C. Bendall, An integrated multi-omic single-cell atlas of human B cell identity. *Immunity* **53**, 217–232.e5 (2020).

96. S. Van Gassen, B. Callebaut, M. J. Van Helden, B. N. Lambrecht, P. Demeester, T. Dhaene, Y. Saeys, FlowSOM: Using self-organizing maps for visualization and interpretation of cytometry data. *Cytometry A* **87**, 636–645 (2015).

97. M. Alcántara-Hernández, R. Leylek, L. E. Wagar, E. G. Engleman, T. Keler, M. P. Marinkovich, M. M. Davis, G. P. Nolan, J. Idoyaga, High-dimensional phenotypic mapping of human dendritic cells reveals interindividual variation and tissue specialization. *Immunity* **47**, 1037–1050.e6 (2017).

98. A. P. Durbin, M. J. Vargas, K. Wanionek, S. N. Hammond, A. Gordon, C. Rocha, A. Balmaseda, E. Harris, Phenotyping of peripheral blood mononuclear cells during acute dengue illness demonstrates infection and increased activation of monocytes in severe cases compared to classic dengue fever. *Virology* **376**, 429–435 (2008).

99. J. L. Kyle, P. R. Beatty, E. Harris, Dengue virus infects macrophages and dendritic cells in a mouse model of infection. *J. Infect. Dis.* **195**, 1808–1817 (2007).

100. V. Upasani, H. T. M. Vo, H. Auerswald, D. Laurent, S. Heng, V. Duong, I. A. Rodenhuis-Zybert, P. Dussart, T. Cantaert, Direct infection of B cells by dengue virus modulates B cell responses in a Cambodian pediatric cohort. *Front. Immunol.* **11**, 594813 (2021).
